# An Outbreak of Synthetic Cannabinoid-Adulterated Tianeptine Products in New Jersey – Case Series

**DOI:** 10.1007/s13181-025-01068-7

**Published:** 2025-03-18

**Authors:** Christopher J. Counts, Anthony V. Spadaro, Trevor A. Cerbini, Alex J. Krotulski, Sara E. Walton, Howard A. Greller, Lewis S. Nelson, Bruce E. Ruck, Oliver Hung, Barry Logan, Diane P. Calello

**Affiliations:** 1 Department of Emergency Medicine, Rutgers New Jersey Medical School, Newark, NJ USA; 2 New Jersey Poison Information and Education System, Newark, NJ USA; 3 Center for Forensic Science Research and Education, Fredric Rieders Family Foundation, Willow Grove, PA USA; 4 Morristown Medical Center, Morristown, NJ USA

**Keywords:** Tianeptine, Cannabinoid, Outbreak

## Abstract

**Background:**

Tianeptine, an atypical antidepressant not approved in the United States, is readily purchased from unregulated markets such as the internet and gas stations. We became aware of a cluster of 34 patients in New Jersey who became ill following ingestion of the tianeptine containing-product Neptune’s Fix, the rate of which (4.6 cases per month) far exceeded the background rate for this substance of 0.5 cases per year.

**Methods:**

We retrospectively identified tianeptine exposures reported to the New Jersey Poison Information and Education System (NJPIES) prior to June 2023 to determine the background rate of tianeptine exposure. From June 2023– February 2024 we prospectively surveilled tianeptine exposures reported to NJPIES, recorded demographic and clinical information, and recruited samples for testing. Six samples of the ingested products were obtained and analyzed using gas chromatography mass spectrometry (GC-MS) and liquid chromatography quadrupole time-of-flight mass spectrometry (LC-QTOF-MS). Whole blood samples from two patients were tested for tianeptine and synthetic cannabinoids.

**Results:**

During the period of interest, NJPIES received 41 exposure calls, with 37 reporting acute toxicity in 34 unique patients, two reporting chronic tianeptine use, and two reporting withdrawal. Among the 37 exposures resulting in acute toxicity, commonly reported effects included altered mental status, tachycardia, hypotension, and seizures. 43% (*n* = 16) were intubated, and 65% (*n* = 24) were admitted to the ICU. Analytical testing of six samples identified variable product composition, containing various xenobiotics including tianeptine, kava alkaloids, natural cannabinoids, and the synthetic cannabinoids MDMB-4en-PINACA and ADB-4en-PINACA. MDMB-4en-PINACA was detected in one of the two patient blood specimens.

**Conclusions:**

These cases represent a marked increase in tianeptine exposures compared with the poison center’s historical average. Analytical testing revealed variable product composition, including the presence of synthetic cannabinoids. Clinicians should be aware that tianeptine containing products are widely available, unregulated, and can be adulterated.

## Introduction

Tianeptine is an atypical antidepressant available as a prescribed medication for the treatment of anxiety and depression in some European, Asian, and Latin American countries. Tianeptine modulates glutamate activity and, at high doses, agonizes the mu opioid receptor [1, 2]. The FDA has not approved its use in the United States (US), and has issued warnings about the risk for dependence, overdose, and death. Nevertheless, tianeptine products with brands names such as ZaZa, Pegasus, Tianna, and Neptune’s Fix are readily purchased online, at headshops or smoke shops, and at gas stations [3, 4].

Calls regarding tianeptine to US poison centers increased from 11 total between 2000 and 2013 to 151 in 2020, and individual poison centers have observed increasing reports over a similar period [3, 5, 6]. The New Jersey Poison Information and Education System (NJPIES) typically receives two or fewer calls per year regarding tianeptine exposure. From June 2023– February 2024, NJPIES received 41 calls regarding tianeptine exposure, the majority of which were about patients who became ill shortly after ingesting purported tianeptine-containing products. As part of our response to this outbreak, we published a report of our preliminary investigation based on the first 20 calls we received that led to the identification of tianeptine products adulterated with natural and synthetic cannabinoids [7]. The aim of this case series was to follow up the outbreak with additional clinical information, including an additional 21 exposures and analytical test results.

## Methods

### Study Population and Data Collection

The investigation was approved by the Rutgers University Institutional Review Board with a waiver of informed consent for research that involves minimal risk. We retrospectively reviewed the NJPIES database (Toxicall^®^, Computer Automation Systems Inc., Colorado) for cases involving tianeptine exposure to establish the background rate, and prospectively surveilled exposures starting at the beginning of the outbreak in June 2023. NJPIES is the sole poison center for the state of New Jersey, serving a population of approximately 9 million. Each call to NJPIES is entered into Toxicall^®^ by a Certified Specialist in Poison Information, producing an electronic record that captures demographic and clinical information, as well as free text descriptions of the exposure history. This information is coded to produce an entry that can be retrieved when the database is queried based on the exposure of interest or other relevant variables. All cases involving tianeptine exposure reported to NJPIES from June 2023– February 2024 were included. Individual records were reviewed for exposure history, demographic and clinical data. The authors classified cases as acute exposure if the patient developed signs or symptoms shortly after a single ingestion of a tianeptine product. Cases were classified as chronic exposures if they were reported as such by the patient or the patient’s healthcare provider. “Withdrawal” was defined as the development of signs or symptoms in the setting of discontinuation or decreased dose of tianeptine in patients who self-identified as chronic tianeptine users.

### Product Sample and Clinical Specimen Collection and Analysis

Six bottles, a mix of open and sealed, labeled “Neptune’s Fix Elixir,” were collected from patients in two reported cases. All six bottles contained a dark liquid and were labeled with an ingredients list containing tianeptine, kava (*Piper methysticum*), magnesium, solvents (propylene glycol, vegetable glycerin, and/or water), and flavoring. Four were cherry flavored (with three different label types) and two were tropical flavored. The samples were sent to the Center for Forensic Science Research and Education (Horsham, PA) for comprehensive chemical analysis of drugs and toxicants. Samples were prepared and dual analyzed using an Agilent Technologies gas chromatograph mass spectrometer (GC-MS) and a SCIEX liquid chromatograph quadrupole time-of-flight mass spectrometer (LC-QTOF-MS). Data were compared against an in-house database containing more than 1,200 targets, including traditional drugs, therapeutics, and novel psychoactive substances [8]. Qualitative confirmation results were determined by comparison to standard reference materials.

Whole blood from two additional patients (different from those who provided product samples) was collected and sent to NMS Labs (Horsham, PA) by the providers caring for the patients. Specimens were tested for tianeptine and 14 synthetic cannabinoids and quantified for tianeptine using liquid chromatography tandem quadrupole mass spectrometry (LC-QQQ-MS).

## Results

**Fig. 1:**
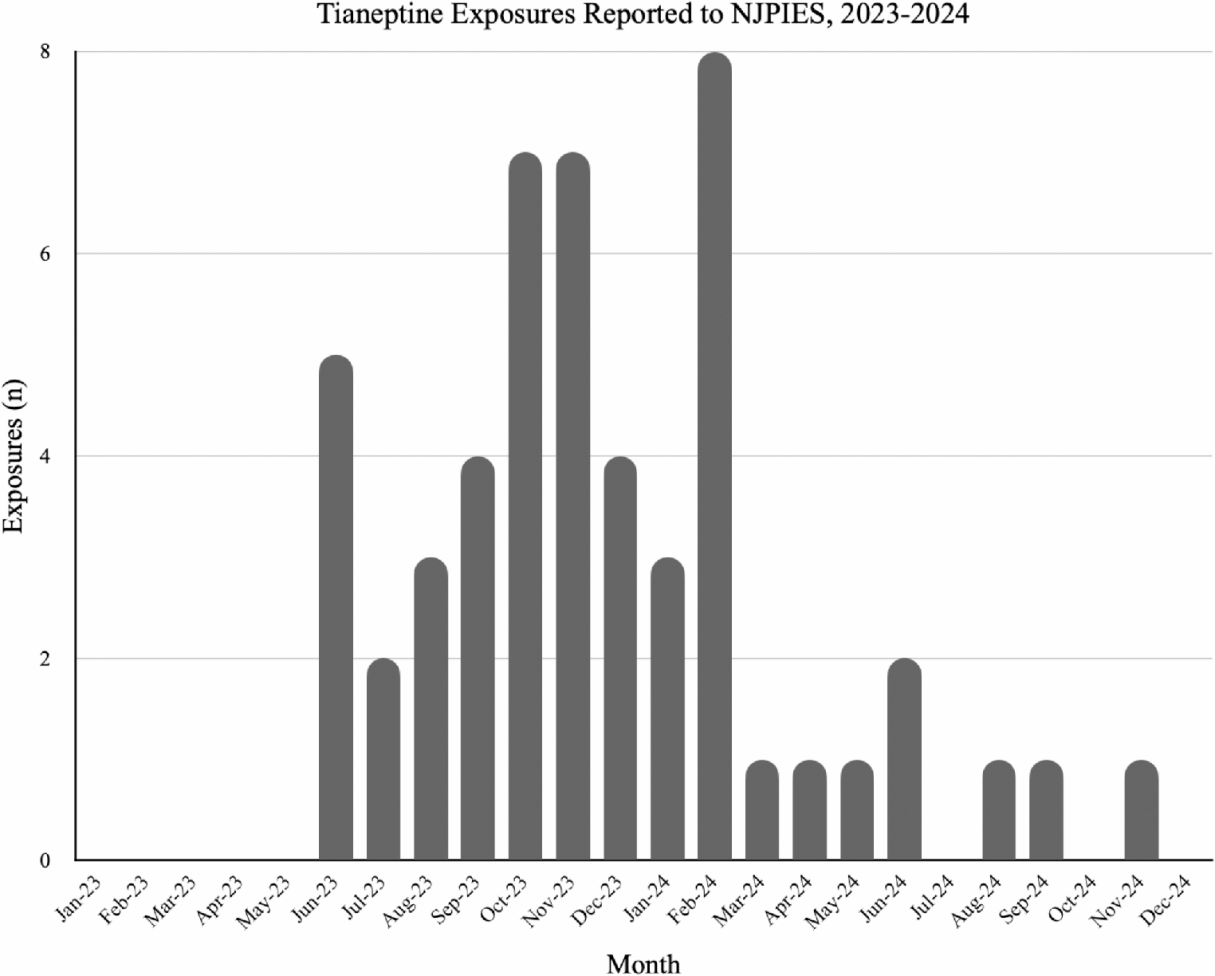
Tianeptine Exposures Reported to NJPIES, 2023–2024

From 2013 to 2022, the average number of tianeptine exposures reported to NJPIES was 0.5 per year. Over the eight-month period of interest, NJPIES received 41 calls regarding tianeptine use from 22 individual hospitals (4.6 cases per month). Of 41 total reports, 37 involved acute effects following purported tianeptine ingestion, two involved chronic tianeptine use, and two involved withdrawal following cessation of chronic tianeptine use. Over the ten months following the period of interest, NJPIES received an average of 0.8 exposure calls per month (Fig. 1).

### Acute Toxicity Following Tianeptine Ingestion

Among the 37 reports of acute illness, 34 unique patients were identified. Three patients presented to the same emergency departments (EDs) on two separate occasions. Patients ranged in age from 19 to 69 years and 71% (*n* = 24) were male. 97% (*n* = 36) were described as having altered mental status. Other common presenting features were tachycardia (*n* = 21, 57%), hypotension (*n* = 15, 41%), seizures (*n* = 15, 41%), and respiratory depression (*n* = 13, 35%). One patient experienced cardiac arrest, with return of spontaneous circulation achieved in the field. There were no fatalities.

Of the 37 exposures, 31 (84%) involved Neptune’s Fix. Reported purchase locations included gas stations, smoke shops, and convenience stores. Co-exposures were reported in 9 of the 37 exposures (24%), and kratom was the most frequently reported (*n* = 5, 14%). Interestingly, one of the Neptune’s fix bottles included a note to “NOT [use] within 12 hours of kratom”, whereas all included cautionary notes about use by pregnant women, use while driving, and use by those under 21 years of age. The demographic and clinical characteristics of the patient encounters are shown in Table 1.

**Table 1:** Demographic and clinical characteristics of patients with acute toxicity

	Value
**Demographic** *n* = 34 patients	
Median age (range), yr	39 (19–69)
Sex, no. (%)	
Male	24 (71%)
Female	10 (29%)
**Clinical — no. (%)** *n* = 37 encounters	
Altered mental status	36 (97%)
Tachycardia (HR > 100)	21 (57%)
Seizure	15 (41%)
Hypotension (< 90 mm Hg systolic or > 15 mm Hg less than patient’s usual)	15 (41%)
Respiratory Depression	13 (35%)
QTc prolongation > = 480 ms	10 (27%)
Agitation	9 (24%)
QRS prolongation > 100 ms	8 (22%)
Mydriasis	6 (16%)
Tremor	4 (11%)
Urinary retention	3 (8%)
Cardiac arrest	1 (3%)
**Selected laboratory abnormalities — no. (%)**	
Hypokalemia (< 3.5 mEq/L)	6 (16%)
Acidemia (pH < 7.3)	5 (14%)
**Therapies — no. (%)**	
Benzodiazepines	20 (54%)
Mechanical ventilation	16 (43%)
Naloxone	12 (32%)
Antipsychotics	4 (11%)
**Disposition — no. (%)**	
Critical care unit	24(65%)
Discharged from ED	9(24%)
Floor	2(5%)

#### Interventions and Outcomes

The most common interventions administered were benzodiazepines (*n* = 20, 54%) and naloxone (*n* = 12, 32%). The reported response to naloxone was variable; two patients responded, three did not respond, and in seven there was an inconclusive response. Among the 37 encounters, 16 (43%) were intubated. 24 (65%) were admitted to the intensive care unit, 2 (5%) were admitted to the floor, and 9 (24%) were discharged from the ED.

#### Analytical Testing

Five of the six bottles were received “open” with the seal broken when first received at the poison center. All six were 10 mL glass bottles and all were labelled as containing tianeptine and kava alkaloids. GC-MS and LC-QTOF-MS analyses are detailed in Table 2. Product composition was variable and included the natural cannabinoids tetrahydrocannabinol (THC) and cannabidiol (CBD), and two synthetic cannabinoids MDMB-4en-PINACA and ADB-4en-PINACA. These unexpected compounds were found in the open and sealed bottles. All bottles contained tianeptine.

**Table 2:** Analytical test results for products or blood from patients with acute toxicity

Patient	Sample or Specimen	Test results^a^	Patient Narratives
1	Neptune’s Fix, open bottle (*n* = 2)	Kavain Tianeptine	Found unresponsive by police after ingesting two bottles of Neptune’s Fix. The police administered naloxone and brought patient to the ED with tachycardia (HR = 180) and tremors. Admitted to the floor and discharged one day later.
1	Neptune’s Fix, sealed bottle	MDMB-4en-PINACA ADB-4en-PINACA CBD THC Tianeptine	
1	Neptune’s Fix, open bottle	MDMB-4en-PINACA ADB-4en-PINACA CBD THC Tianeptine	
2	Neptune’s Fix, open bottle (*n* = 2)	Kavain Tianeptine	Found with altered mental status, slurred speech, and surrounded by empty bottles of Neptune’s Fix. Became obtunded, was intubated by EMS, seized, and had dilated pupils. Computed tomography imaging of the brain was unremarkable. Extubated the next day and discharged after two days.
3	Blood	Tianeptine 460 ng/mL MBMB-4en-PINACA detected	Found seizing in car with bottles of Neptune’s Fix, Zaza tianeptine, and kratom. EMS described the patient as “presenting like an opioid and cocaine overdose at the same time.” In the emergency department, patient had multiple seizures and was intubated. Extubated the next day and reported ingesting Neptune’s Fix.
4	Blood	Tianeptine 89 ng/mL Synthetic cannabinoids not detected	Ingested Neptune’s Fix while idling in a parking lot, and subsequently crashed vehicle into building. Seizing upon EMS arrival, followed by a period of agitation, for which EMS administered intramuscular midazolam. Intubated on arrival to the ED. Extubated later the same day.

^a^ = qualitative testing for plant and synthetic cannabinoids, quantitative testing for tianeptine in whole blood specimens

Whole blood specimens from two additional patients different from those who supplied product samples were quantitatively tested for tianeptine and qualitatively tested for 14 synthetic cannabinoids. In one patient, the tianeptine concentration was 460 ng/mL (therapeutic range: 278–366 ng/mL), and MDMB-4en-PINACA was detected [9, 10]. In another patient, the tianeptine concentration was 89 ng/mL and none of the synthetic cannabinoids tested for were detected.

### Chronic Use and Withdrawal

Two cases involved the chronic use of tianeptine. One patient presented with chest pain, and one with abdominal pain. Both patients attributed their symptoms to chronic tianeptine use. Two patients reported withdrawal following cessation of chronic tianeptine use. In both cases, the patients reported large daily ingestions of tianeptine (7 g and 13.5 g total). When prescribed for depression, standard daily doses are 25–50 mg [2]. One patient self-tapered to a lower daily dose and was admitted for inpatient detoxification. One patient abruptly ceased tianeptine use, and developed a withdrawal syndrome consistent with opioid withdrawal, characterized by piloerection, vomiting, diarrhea, and dilated pupils. These findings improved with buprenorphine, clonidine, and lorazepam.

## Discussion

This case series describes an outbreak of 41 cases of tianeptine exposure reported to NJPIES, our subsequent identification of synthetic cannabinoids in ingested drug product samples, qualitative and quantitative identification of tianeptine in the blood of two patients, and qualitative confirmation of MDMB-4en-PINACA in the blood of one affected patient. Common clinical effects in this cluster of acute toxicity were altered mental status, respiratory depression, tachycardia, hypotension, and seizure. Intubation and admission to the ICU were common, and most patients recovered without major sequelae following a short hospitalization.

The severity of these clinical findings may reflect tianeptine effects, synthetic cannabinoid toxicity, polysubstance ingestion, or a combination. Acute effects from tianeptine were likely contributory in some cases; one of the patients with measured tianeptine blood concentrations had a level higher than what is considered a therapeutic range for depression of 278–366 ng/mL [9, 10]. Although frequently described as a tricyclic antidepressant, recent literature suggests the unique structure of tianeptine results in a mechanism distinct from tricyclic antidepressants, including mu opioid receptor agonism, and modulation of glutamate and dopamine signaling [1, 2, 11]. Many of the patients in this series demonstrated respiratory depression, an expected effect from tianeptine’s mu opioid receptor agonism. While tachycardia has been reported in tianeptine exposures, hypotension is less consistently reported [5, 6].

The agitation and seizures observed in this cluster are consistent with prior reports of synthetic cannabinoid toxicity [12]. In the late 2000s, synthetic cannabinoids emerged as novel drugs [13, 14]. Subsequently, there have been several notable outbreaks of synthetic cannabinoid toxicity, including multiple cases of acute kidney injury following use of XLR-11 in 2012, agitation from AMB-CHMINACA in 2014, and “zombie-like” catatonia from AMB-FUBINACA in 2016 [15–17]. Clinical effects vary based on the individual compound, although common findings include altered mental status, seizures, psychosis, and end-organ toxicity such as acute tubular necrosis, hepatotoxicity, and cardiotoxicity [12, 18].

MDMB-4en-PINACA belongs to a new generation of synthetic cannabinoids that are characterized by the presence of an alkene tail and were first identified in drug material from European prisons in 2017 [19, 20]. In-vitro analysis of CB1 receptor activation showed MDMB-4en-PINACA to be the most potent of this group of compounds [20]. ADB-4en-PINACA has been detected in a plant-like drug material in the United States, drug papers seized from a Scottish prison, in the hair of a prisoner in China, and recently in paper strips recovered from a jail in the United States; however, its clinical toxicity is scantly described in the literature [21–24].

While the majority of the calls received were for acute effects, two cases of potential withdrawal highlight the risk of tianeptine dependence. In both cases, the patients used large doses of tianeptine daily and exhibited a withdrawal syndrome when the tianeptine was either discontinued or the dose decreased. One patient’s withdrawal was managed successfully with several doses of buprenorphine, clonidine, and lorazepam. Literature on the management of tianeptine withdrawal is limited to case reports; buprenorphine microdose induction and methadone have been used [25, 26]. Whether withdrawal of tianeptine is a unique syndrome, results from exacerbation of anxiety or depression, or is related to changes in neurotransmitter signaling from physiological dependence remains undefined. Tianeptine withdrawal is likely to become an increasingly relevant issue for patients and clinicians; at least twelve states have scheduled or restricted sales of tianeptine, a number that is expected to increase as the effects of tianeptine gain greater attention [27, 28].

The broader use of tianeptine highlights the regulatory challenges associated with novel and unscheduled drugs. Tianeptine is not approved for use by the FDA, is not scheduled at the federal level, and is not subject to the rules regulating analogues of scheduled substances. As a result, such products are often made available to consumers in the form of dietary supplements or research chemicals [29]. The FDA “considers tianeptine to be a substance that does not meet the statutory definition of a dietary ingredient and is an unsafe food additive” [4]. Despite this, some tianeptine products are marketed in this manner. The Neptune’s Fix bottles we obtained all were flavored yet bore the label “for research purposes only” alongside the descriptor “fast acting,” leaving little ambiguity regarding its intended use.

This study has several limitations. First, reporting to poison centers is voluntary, and our reported cases likely under-represent the number of cases of acute toxicity and withdrawal that occurred during this period. The completeness and accuracy of the clinical data collected is dependent on the reporting of the caller, completeness of the information available to them, and the poison specialist and/or toxicologist receiving the call. For example, co-ingested substances that were not known or reported may have been present in some cases. Second, we were able to obtain confirmatory analytic testing on only a small number of the total cases, and contents identified in these product samples may not be generalizable to all patients in the outbreak. For cases with blood specimen analysis, the presence of 14 synthetic cannabinoids was tested, which is a small fraction of the hundreds of synthetic cannabinoids that exist and limits the ability to generalize these test results to other patients in the outbreak.

## Conclusion

This case series describes an outbreak of 41 cases of tianeptine exposure reported to NJPIES from June 2023– February 2024, a marked increase compared with the poison center’s historical average. The majority of these reports described acute toxicity, and our subsequent investigation led to the identification of tianeptine products adulterated with natural and synthetic cannabinoids. From November 2023 to February 2024, the FDA issued several warnings to consumers “not to purchase or use any Neptune’s Fix products, or any other product with tianeptine,” and sent letters to retailers urging them to stop selling Neptune’s Fix and other tianeptine-containing products. From January to February 2024, two businesses issued voluntary recalls of all Neptune’s Fix products [30]. Overall, this outbreak highlights tianeptine and emerging synthetic cannabinoids as substances of public health concern, as well as the importance of regional poison centers as agents of surveillance for novel outbreaks.

## Declarations

### Previous Presentation

Data in this manuscript were previously presented at ACMT’s Annual Scientific Meeting, Washington, D.C., 2024.
